# Unsupervised machine learning models reveal predictive markers of glioblastoma patient survival using white blood cell counts prior to initiating chemoradiation

**DOI:** 10.21203/rs.3.rs-2834239/v1

**Published:** 2023-04-21

**Authors:** Wesley Wang, Zeynep Temerit Kumm, Cindy Ho, Ideli Zanesco-Fontes, Gustavo Texiera, Rui Manuel Reis, Horacio Martinetto, Javaria Khan, Mark D. Anderson, M Omar Chohan, Sasha Beyer, J Brad Elder, Pierre Giglio, José Javier Otero

**Affiliations:** The Ohio State University Wexner Medical Center; The Ohio State University Wexner Medical Center; The Ohio State University Wexner Medical Center; Barretos Cancer Hospital; Barretos Cancer Hospital; Barretos Cancer Hospital; Fundación para la Lucha contra las Enfermedades Neurológicas de la Infancia; University of Mississippi Medical Center; University of Mississippi Medical Center; University of Mississippi Medical Center; The Ohio State University Wexner Medical Center; The Ohio State University Wexner Medical Center; The Ohio State University Wexner Medical Center; The Ohio State University Wexner Medical Center

**Keywords:** Glioblastoma, Survival Outcomes, Clinical Decision-making, Machine Learning

## Abstract

**Purpose:**

Glioblastoma is a malignant brain tumor requiring careful clinical monitoring even after primary management. Personalized medicine has suggested use of various molecular biomarkers as predictors of patient prognosis or factors utilized for clinical decision making. However, the accessibility of such molecular testing poses a constraint for various institutes requiring identification of low-cost predictive biomarkers to ensure equitable care.

**Methods:**

We collected retrospective data from patients seen at Ohio State University, University of Mississippi, Barretos Cancer Hospital (Brazil), and FLENI (Argentina) who were managed for glioblastoma—amounting to nearly 600 patient records documented using REDCap. Patients were evaluated using an unsupervised machine learning approach comprised of dimensionality reduction and eigenvector analysis to visualize the inter-relationship of collected clinical features.

**Results:**

We discovered that white blood cell count of a patient during baseline planning for treatment was predictive of overall survival with an over 6-month median survival difference between the upper and lower quartiles of white blood cell count. By utilizing an objective PDL-1 immunohistochemistry quantification algorithm, we were further able to identify an increase in PDL-1 expression in glioblastoma patients with high white blood cell counts.

**Conclusion:**

These findings suggest that in a subset of glioblastoma patients the incorporation of white blood cell count and PDL-1 expression in the brain tumor biopsy as simple biomarkers predicting glioblastoma patient survival. Moreover, use of machine learning models allows us to visualize complex clinical datasets to uncover novel clinical relationships.

## Introduction

Glioblastoma (GB) patients suffer from aggressive solid tumors of the central nervous system, with 95% projected to be deceased within 5 years following diagnosis (1). Cause of death in glioblastoma varies, but may include herniation secondary to mass effect, treatment complications, and aspiration pneumonia due to brainstem dysfunction (2). Despite aggressive adjuvant therapy, however, almost all patients will experience tumor progression. Nevertheless, major strides have been made within neuro-oncology to merge personalized medicine to better predict patient outcomes. Although therapies for GB management after maximal-safe surgical resection have remained largely unchanged since the concomitant use of temozolomide (TMZ) with radiation (ChemoRT), molecular biomarkers have been integrated as critical tools in the diagnosis and prognostication of gliomas (3–4). From a diagnostic standpoint, lack of an *IDH*-mutation defines the current World Health Organization (WHO) 2021 definition of a GB (4). Moreover, *TERT* promoter mutations, EGFR amplifications, and modifications of chromosome 7/9/10 are all recognized molecular changes present in GB.

Prognostically, several molecular markers have been suggestive of predicting outcome in GB patients. Namely, *MGMT* promoter methylation status is routinely tested to predict efficacy of temozolomide treatment due to the antagonistic role of MGMT in temozolomide DNA alkylation (5–6). However, other markers such as CDKN2A/B loss and EGFRvIII have been shown in retrospective studies to act as poor prognostic markers in GB patients or subset populations (7–8). The need for prognostic markers of disease is critical in ascertaining whether certain patients should be monitored more closely during follow-up. Furthermore, the logistics of efficiently scheduling patients between visits with neuro-oncology, neurosurgery, neuroradiology, and radiation oncology would significantly benefit from improved triaging methods that prioritize the most sensitive patients. Unfortunately, the accessibility of molecular markers is not equal across healthcare ecosystems (9–10). Current discussions in the field of neuro-oncology have pointed to major accessibility barriers and bioethical implications of pure reliance of molecular biomarkers of disease to understand cancer (10). Molecular pathology testing requires batching to reduce patient costs, a need that results in significant costs in turn-around-time for molecular assays (11). In consequence, groups have sought ways to evaluate outcomes of glioma patients using surrogate measures such as neuro-cognitive testing, psychiatric examination, or image analysis of histology to predict molecular phenotypes as these turn-around-times are superior to those of molecular pathology (9, 12–13). While promising however, integration of these approaches will take time whereas routinely collected data is readily available.

To better explore potential prognostic markers already routinely collected while managing GB patients, we retrospectively evaluated 581 patients at four sites from three countries: Ohio State University (US), University of Mississippi (US), Barretos Cancer Hospital (Brazil), and FLENI (Argentina). Patient features were assessed using an unsupervised learning approach to understand how clinical metrics related amongst each other—including relevant clinical endpoints such as progression-free survival (PFS) and overall survival (OS). Furthermore, by utilizing these novel relationships, we better characterized the relevance of routine complete blood counts (CBCs) and merged its utility with routine IHC staining in pathology to suggest novel workflows which may predict outcomes of patients with GB.

## Methods

### Selection Criteria and Clinical Data Collection

Evaluation of retrospective clinical charts from the electronic health record (EHR) was performed at The Ohio State University (OSU) with IRB approval under study number 2020C0062, University of Mississippi (UMMC) with IRB approval under study number UMMC-IRB-2022–93, Barretos Cancer Hospital with IRB approval under study number 1604/2018, and FLENI under ethics committee/patient’s informed consent approval. Clinical records from patients receiving GB care from 2012 to 2020 were evaluated. Inclusion criteria for patients were designated by prior history of GB treatment. We however, excluded patients with prior history of low-grade gliomas, IDH-mutation, or patients with external care prior or following contact which led to poor documentation of disease course. Study data was collected and managed using REDCap electronic data capture tools (14–15). REDCap was utilized to (a) securely store and deidentify patient records for downstream use, (b) ensure consistency of data collection, and (c) be distributable to collaborators desiring to replicate or collect similar metrics as described in the study (**Supplementary Document 1**).

Clinical features were collected as listed from the electronic health record. Comorbidity scoring was performed by manually listing known comorbidities and scoring them in REDCap following the Charlson Comorbidity Index (CCI) (16). Lesion and molecular features were extracted from radiology and pathology reports, respectively. Results from complete blood counts (CBCs)—including white blood cell count (WBC), neutrophil count, lymphocyte count, and platelet count were designated as CBC draws occurring approximately 2–4 weeks after surgery during patient follow-up with neuro-oncology prior to beginning ChemoRT. Neutrophil-to-lymphocyte ratio (NLR) was calculated by dividing the neutrophil count against lymphocyte count in a patient. Steroid dose was defined as the total daily steroid intake during the same day as CBC collection. Variables containing dates were converted to deidentified values of time to remove potential patient timeline identification after calculating relevant timespans in days. OS in the study was defined as the time from primary tumor resection until time of death. PFS was defined as time from primary tumor resection until time of initial detection of a novel enhancing lesion on imaging. Confirmation of enhancement as being either cancer recurrent or treatment reactive in nature was completed by clinical correlation and consensus from a multi-disciplinary tumor board.

### Exploration of Clinical Features

Collected clinical data from REDCap was exported and deidentified for use in R. Missingness of data was evaluated using the naniar package (17). Cases with over 30% missing records were not evaluated using unsupervised analysis while any missing features in the remainder of cases were imputed using the mice package in R (**Supplementary Fig. 1**; 18). Using the factoextra package, principal component analysis (PCA) was performed over the dataset and relations of clinical features were visualized using eigenvector plotting of our PCA as described in our previous work (19–20).

Exploration of clinical features as a function of OS was explored using cox regression modeling with non-imputed data. Clinical features were independently evaluated using a univariate cox model to assess prediction of overall survival time. P-values less then 0.05 were omitted in subsequent modeling. Univariate-significant features were then evaluated using a multivariate cox regression model using patient records found to have no missing relevant features. Patient survival was further explored by quartile stratification of patients for relevant clinical features (**Supplementary Table 1**). Highest and lowest 25% groups were compared for progression-free survival and overall survival using the survival and survminer packages (21–22). Visualization of relevant clinical confounders was performed using R with ggplot2 (23).

### PDL-1 Image Analysis

PDL-1 immunohistochemistry imaging was collected from relevant patient samples at Ohio State which underwent routine PDL-1 evaluation at time of primary surgery for GB. Image tiles were collected from digital pathology slides and processed in R using EBImage (24). RBG images were deconvoluted for hematoxylin and DAB stain layers using scikit-image and reticulate (25–26). Segmentation for either hematoxylin or DAB staining was performed using Otsu thresholding (27). Segmented regions were calculated for morphologic features using EBImage. Filtration of segmentation was performed using a random-forest based classifier trained over intensity-based morphology features to classify segmentation as no stain, low stain, medium stain, and high stain (**Supplementary Fig. 2;** 28). No stain segmentations were filtered. PDL-1 staining was represented by the ratio of DAB-stained pixel to hematoxylin-stained pixels—calculated from segmentation areas in EBImage based upon objective stain scoring approaches delineated by Igarashi et al (29).

## Results

### Patient Characteristics

**Figure 1:**
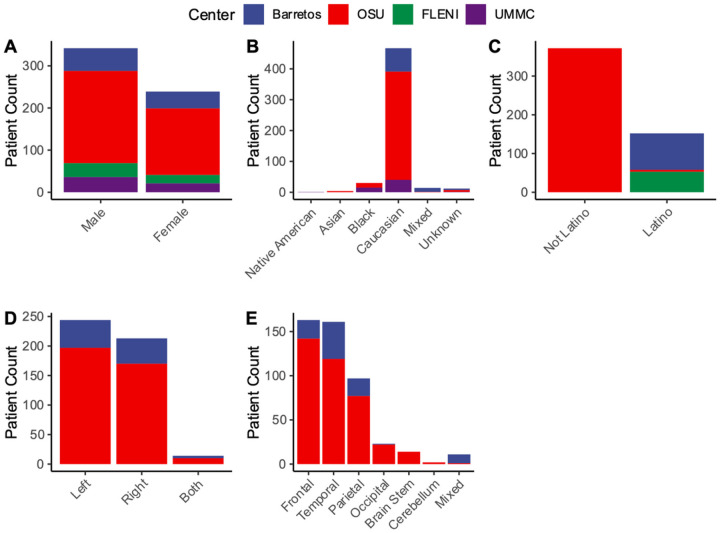
Distribution of patient demographics and lesion characteristics with respect to center. Bar plot distributions of (A) gender, (B) race, (C) ethnicity, (D) lesion sidedness, and (E) lesion lobe location across collected centers. Centers are represented as OSU (red), UMMC (purple), Barretos (blue), and FLENI (green). Omission of center in plot indicates variable was not collected.

A total of 581 patients were evaluated and documented in REDCap across four centers. In turn, overall demographic scores and biases across centers were assessed. Overall, mean age was 61 years [range: 20–89] (Table 1). Males slightly exceeded females in representation across centers (Fig. 1A). The distribution of ethnic origin directly related to the site of collection. North American sites represented predominately Caucasians and Black/African Americans with non-Latino ethnicity, while South American sites were of Caucasian and mixed ancestry with Latino origin (Fig. 1B–C). All patients had biopsy confirmed GB with resection of tumor at primary surgery. There was a slight predominance of left sided lesions primarily occurring in the frontal, temporal, and parietal lobes across sites (Fig. 1D–E). On average, mean tumor diameter was 4.34 cm [range: 0.1–9.5] with most without midline shift on imaging (Table 1). It was however noted that several clinical features were not collected in sites outside OSU (**Supplementary Fig. 1**). When tested, IHC studies showed primarily ATRX locus intact (98%) and p53 mutation (78%) as defined by positivity in over 10% of cells. Predominant molecular features were *EGFR* amplification (56%) and unmethylated *MGMT* promoter status (57%).

**Figure 2:**
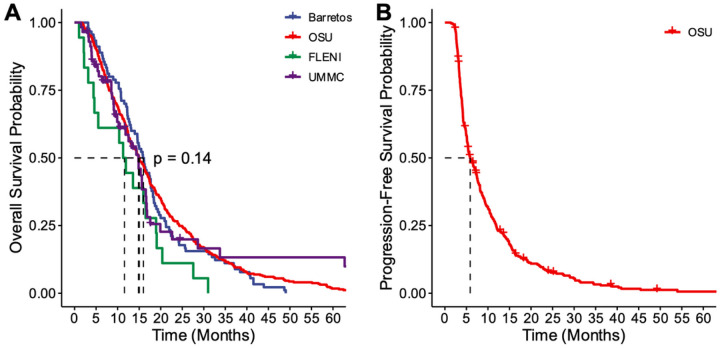
Overall survival and progression free survival across sites. (A) OS and (B) PFS of patients across sites. Centers are represented as OSU (red), UMMC (purple), Barretos (blue), and FLENI (green).

ChemoRT overall followed a traditional 60 Gray-30 fractions radiation plan, but some patients received hypo-fractionated regimen or did not complete ChemoRT (Radiation Plan: 55.59 [range: 5.34–75]; Radiation Fractions: 26.8 [range: 1–50]). Adjuvant TMZ therapy was completed at 3 cycles on average [range: 0–19]. Symptomatic management of edema with steroids at time of CBC collection was on average 2.84 mg/day with a broad range of use [range: 0–24]. Outcomes of patients were assessed as both PFS and OS from date of primary surgery. Among centers, median OS ranged from 12–16 months without significant difference among groups while median PFS was 6 months at OSU (Fig. 2).

#### Unsupervised Analysis of Patient Reveals Capabilities of CBCs in Predicting Overall Survival Outcome and Time to Enhancement

**Figure 3:**
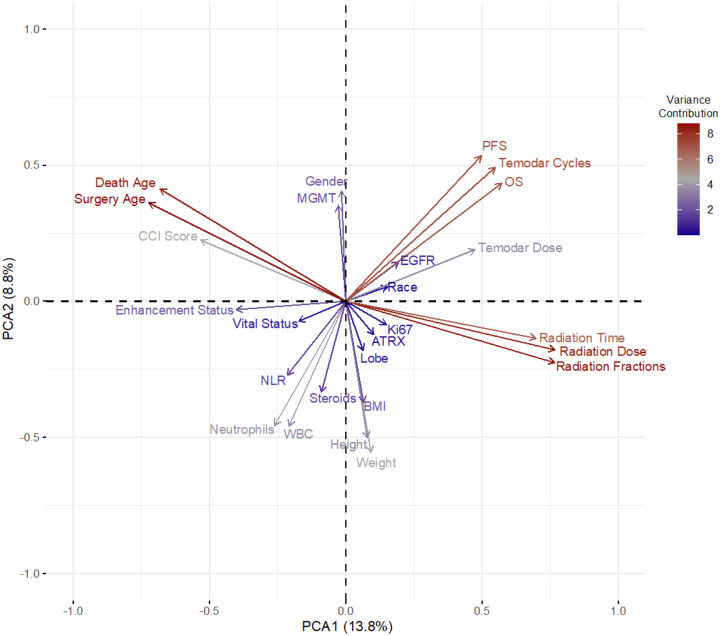
Eigenvector analysis of clinical features. Collected clinical features from Redcap data was assessed for all patients with less than 30% of data missing (n = 376). Missing data was imputed in R. Contribution to data variation is highlighted by the color legend and distance of a vector from the origin. Relationship of features are represented by directionality with direct relationships represented by same directionality, inverse relationship by opposite directionality, and no relationship as orthogonal directionality. Omitted features did not have high enough variance contribution to visualize.

Utilizing the various collected clinical datapoints, we sought to evaluate whether specific clinical features were correlated with relevant outcomes in patients that have not been previously integrated in clinical practice. Specifically, we posited that applying an unsupervised machine learning approach could represent clinical relationships amongst features to allow us to visualize novel findings predictive in GB patient prognosis. To do so, our multidimensional dataset was reduced using principal components analysis (PCA) to stratify patients by these clinical metrics and visualized using PCA eigenvector plotting (Fig. 3). The directionality of eigenvectors (arrows) amongst other eigenvectors represents direct correlations through same arrow directionality, inverse correlations through opposite directionality, and no correlation through orthogonal directionality. With these considerations, we identified relationships of features to relevant clinical outcomes. Notably, the directionality of eigenvectors for OS and PFS occurred similarly [upper right quadrant] with inverse directionality to CBC-related measures—notably WBC and neutrophil measure in the lower left quadrant (Fig. 3). In contrast, enhancement status was shown on the left side of the plot, but other vectors had less robust directional relationships—with the most prominent being inverse directionality of adjuvant TMZ dosage in the upper left quadrant. Nevertheless, as our eigenvector plot uncovered novel variations amongst clinical features, we further explored these findings using predictive modeling.

Cox regression modeling was applied to predict OS based on our clinical features. Based on our PCA eigenvector plot, we theorized that CBC related metrics would be significant to predicting survival time. Univariate models were first performed over the study population to assess which features were found to have significance. In total, 11 separate features were found to be significant [Patient Age, CCI score, *MGMT* methylation status, WBC count, Neutrophil count, NLR, Radiation Dose, Radiation Fractions, Overall Radiation Time, Adjuvant TMZ Cycles, and Adjuvant TMZ Dose] (Table 2). Taking these features, a multivariate cox regression model was constructed to then assess which features independently contribute significantly to predicting survival outcome. Only 5 clinical features were found to be relevant [Patient Age, *MGMT* methylation status, Neutrophil count, Radiation Dose, and Adjuvant TMZ Cycles] (Table 3). While the contribution of neutrophil count was significant in our multivariate model, the hazard ratio (HR) was small [1.064 (1.013–1.117)]. In consequence, although a small, but significant risk to poorer survival was evidenced by increased neutrophil load, we next sought to explore causes to the observed difference our cox modeling showed against our PCA eigenvector plot.

**Figure 4:**
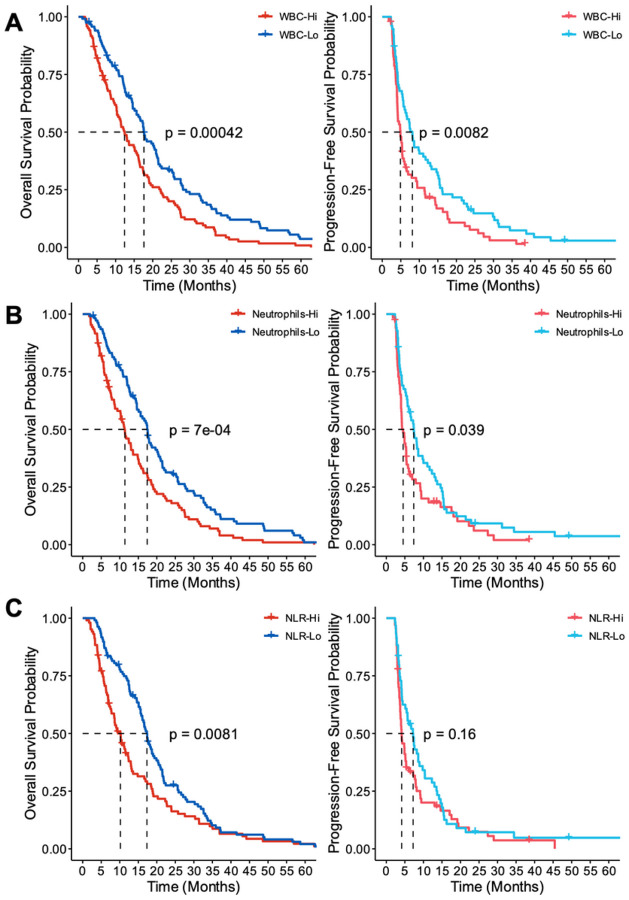
KM curve survival outcomes based upon CBCs. OS (left) and PFS (right) of patients in upper 25% [red] and lower 25% [blue] of cases stratified based on (A) WBC count (n=186/group), (B) Neutrophil count (n=212/group), and (C) Neutrophil-to-Lymphocyte ratio (n=217/group). All CBCs were collected prior to initiating ChemoRT as routine baseline by neuro-oncology.

While cox regression modeling validated the assumption that CBCs have predictive capabilities to survival time, we further examined the discordance of our strong correlations seen in the PCA eigenvector plot against the smaller HRs calculated in our cox models. Specifically, we hypothesized the differences seen in our results may be underscored by robust survival differences present in the extremes of our CBC metrics. Specifically, based on our univariate cox results, we explored survival differences in patients when stratified by WBC count, neutrophil count, and neutrophil:lymphocyte ratio. CBC measures were evaluated by stratifying populations into quartiles with the lower 25% (lo in blue) and upper 25% (hi in red) of patients evaluated. In both overall WBC load and neutrophil load, PFS was significantly worse in the hi group relative to the lo group (**p = 0.0082 and p = 0.039**, respectively) (Fig. 4A–B). Furthermore, evaluation of OS using WBC load and neutrophils showed similar trends between groups (**p = 0.00042 and p = 0.0007**, respectively). However, while NLR did show significant survival difference in OS (**p = 0.0081**), the difference in PFS between groups did not reach our threshold of significance (Fig. 4C). Combined with the previous analyses, our findings strongly support the use of CBCs in predicting both OS and PFS in patients. Specifically, the evaluation of routinely drawn WBC and neutrophil count at time of pre-ChemoRT planning is shown to be correlative with poorer survival outcomes in patients with high load compared to patients with low load.

### WBC Load is Reflective of Intrinsic Tumor Microenvironment Changes Present in Glioblastoma

**Figure 5:**
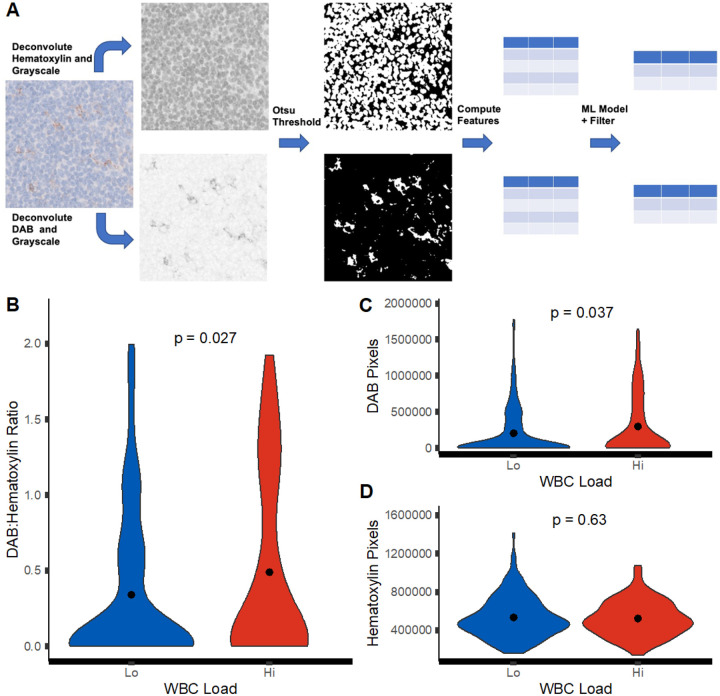
Assessment of PDL-1 expression in relation to WBC load. (A) Workflow for automatic PDL-1 signal quantification and morphology calculation. Violin plots of (B) DAB to Hematoxylin pixel ratio, (C) DAB pixel, and (D) Hematoxylin pixel distribution in WBC-hi and WBC-lo groups.

As the measures of WBC load evaluate circulating immune counts, we posited these differences in peripheral immune activity may correlate with intrinsic tumor microenvironment differences found in primary GB events. To assess this, we selected PDL-1 IHC staining done in a subset of patient during clinical evaluation. Amongst our study population, 57 cases had been evaluated by neuropathology for PDL-1 and representative images were collected from cases and segmented using computer vision to quantify staining (Fig. 5A). Due to the membranous staining of PDL-1 however, staining was quantified as the ratio of DAB-positive PDL-1 staining against hematoxylin nuclear staining to control for cellularity of tissue. In turn, increased detection of PDL-1 staining is represented by an increased ratio. With this approach, we predicted the detection of PDL-1 staining ratio to be elevated in the WBC-hi group due to the associated poor prognostic outcome of increased PDL-1 staining. In fact, it was observed that the WBC-hi group showed higher ratios of PDL-1 DAB to hematoxylin staining when compared to WBC-lo (**p = 0.027**, Fig. 5B). Furthermore, assessing the two sides of the ratio comparison it was seen that while the amount of DAB pixels detected in an image was higher in the WBC-hi group (**p = 0.037**), the detection of hematoxylin pixels did not vary between groups (**p = 0.63**) (Fig. 5C–D). In conclusion, it could be inferred the increased detection of PDL-1 staining ratio in the WBC-hi group was not a product of increased cellularity as the distribution of hematoxylin was not different. Overall, these findings infer that an increase in PDL-1 staining was correlated with the WBC-hi group which showed poorer survival outcomes in patients—validating that the observed differences in WBC load are correlative to initial immune activity present in GB lesions at resection.

### Steroid Tapering is Highly Heterogenous Following Surgery and May Influence WBC Load

**Figure 6:**
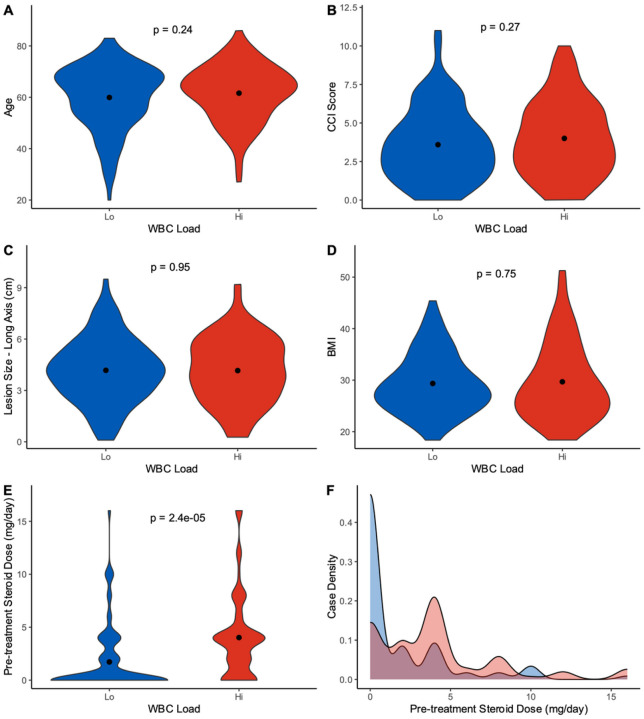
Steroid tapering varies amongst WBC-hi/lo groups. Groups were compared between (A) patient age, (B) CCI score, (C) lesion size, (D) BMI, and (E) steroid dosing at time of CBC collection using violin plots. (F) Distribution of daily steroid intake is visualized using a density plot between groups.

Although our analyses found strong correlation of CBC load to survival outcomes, we further evaluated potential clinical confounders which may influence CBC levels prior to ChemoRT. As shown in the analyses of our study population, heterogeneity in patient demographics, lesion characteristics, and patient management was present (Table 1). In turn, an assessment to identify whether specific clinical features significantly varied between our hi and lo populations was critical. As patient age, CCI score, and *MGMT* methylation status were predictive of survival in our univariate cox model, we assessed these factors in addition to other clinical features that were shown to be correlative to CBC measures in the eigenvector plot [lesion size, steroid intake, and BMI] (Fig. 3**&** Table 2). Comparing WBC-hi and lo groups, *MGMT* methylation status distribution was not found to significantly vary between groups (X^2^ = **0.88, p-value = 0.35**). However, while patient age, CCI score, lesion size and BMI were found to not vary between groups, a significant variation in steroid dosing between groups was present (**p = 2.4e-05**), with the WBC-hi group showing a higher mean daily steroid intake compared to those in the lo group (Fig. 6A–E). Evaluating the distribution of steroid doses given to patients at time of post-surgical CBC, most patients received low doses of steroids less than 6mg a day (Hi: 76.9%; Lo: 89.5%); however, a larger percent of patient from the WBC-lo fully tapered off steroids (Hi: 24.2%; Lo: 65.1%). Nevertheless, assay of patient distribution shows 55.9% of patients across both groups remain on steroids at follow-up prior to initiating therapy (Fig. 6F). Overall, this finding highlights the additional confounding role steroid intake may have on the survival differences seen between CBC load, but also emphasizes the heterogeneity of steroid dosing in patients following GB resection.

## Discussion

### PCA Eigenvector Visualization Can Better Uncover Clinical Relationships in Complex Clinical Datasets

With the adoption of the EHR, patient related data has exponentially grown but remains an under-used resource due to the complexity of mining data and uncovering novel associations. It has been commented that such data provides potential for generating new patient stratification strategies (30). In turn, our unsupervised PCA eigenvector approach may help rectify these challenges by providing easy to interpret visualizations of clinical data relationships. Illustrating this method utility in our study, the vectors measuring radiation metrics showed opposite directionality to features measuring patient age. The directionality of these vectors thus underscores the known clinical management of GB patients whereby older individuals often receive hypo-fractionated radiation dosage due to toxicity (31). In turn, the application of our eigenvector plot to initially detect relationships of patient survival to CBCs illustrates the utility of applying this method in large clinical datasets as a first pass visualization approach in identifying novel relationships to explore in a clinical study. As the framework of this approach is built on existing open-source packages, the deployment of this model as a clinical research tool is highly realistic.

### CBC Stratification Can Be Applied to Identify Patients with Poorer Predicted Survival Outcome

In our study, it was highlighted that CBC metrics—namely WBC and neutrophil count—were inversely related to OS and PFS. Interestingly, past studies have indicated that several components of CBC tests have predictive outcomes in OS. Namely, Pierscianek et al. and Jarmuzek et al. both retrospectively identified similar effects of WBC counts as prognostic factors in OS using CBCs collected during admission or pre-operatively for a potential glioma (32–33). Nevertheless, these studies do not recapitulate the relevant timepoint within our study which suggested WBC counts collected prior to initiating ChemoRT as most predictive to survival. To this point, Schernberg et al. similarly assessed the utilization of CBCs during pre-treatment for ChemoRT and found neutrophilia, advanced age, and more complete resection as features which independently decreased OS in a multivariate model, while steroid consumption did not (34). These findings largely parallel our cox regression models which found patient age, ChemoRT treatment regime, and neutrophil count as predictive to OS. Furthermore, it was noted as well that the median survival time in the WBC-hi group was approximately 12 months while the WBC-lo group was 18 months. Although the coverage of patients in our study ranges the past decade, studies have reported the median survival of GB ranging from 12 to 15 months with ChemoRT treatment (35–37). In turn, it may be suggestive that patients with lower WBC load, particularly with regards to neutrophil count, experience better survival outcomes than just those with neutrophilia declining more rapidly.

Outside of OS however, our data similarly indicated that WBC load plays a potential role in PFS. While less studies have explored the implication of CBCs to progression outcomes, data has suggested lymphopenia as reducing PFS in primary lesions and use of CBCs including platelet, lymphocyte, and eosinophil counts as predictive in measuring performance outcomes in recurrent GB patients (38–39). Although these CBC-specific findings weren’t recapitulated in our studies, our current findings and past research support the notion that CBC measures are prognostic markers in assessing OS and PFS. In turn, initial pretreatment planning may weigh consideration of these metrics to assist in determining which patients should be more carefully followed after treatment.

### Fast Tapering of Steroids following Surgery May Influence Long Term Outcomes of Patients

An important consideration that should be highlighted in our study was the evidence that prior to ChemoRT treatment, large distribution in steroid tapering across our study groups was present. Namely, while our study and others have evidenced that WBC prediction is independent of corticosteroid use statistically, the biologic influence of corticosteroids to both neutrophil and lymphocyte count has been long recognized (40–41). Alternative studies, like Dubinski et al., have directly implicated that administration of dexamethasone induces leukocytosis which was associated to poor survival (42). Although steroids have been long used to provide supportive therapy, these findings may suggest the need to set a more consistent standard of fast tapering patients off steroids (43). Our observed WBC counts and PDL-1 measure may be suggestive of which patients in fact need to have steroid doses modified due to influence on immune cell activity. Studies have evidenced that the increased administration of steroids promotes immune cell dysfunction, namely in T-cell compartments, by promoting increased expression of PDL-1 in the microenvironment that advances dysregulation of immune response (44–45). Although these findings may complicate the utilization of CBC measures for survival prediction, these observations more importantly underscore the need to better consider the use of steroids for the symptomatic relief of GB patients.

## Conclusion

The use of data derived from the electronic health record will remain a powerful resource in spite of the growing popularity of molecular testing due to the challenges in molecular testing deployment. Moreso, challenges in exploring clinical data from the electronic health record have not been a consequence of non-significant data, but rather the sheer complexity of both collecting and assaying data to uncover novel research discoveries. The deliverables of our study include our standardized REDCap data collection form for evaluation of GB, unsupervised analysis framework to initially explore clinical datasets through PCA eigenvector visualization, and automated image analysis pipeline for PDL-1 staining. Furthermore, the measure of CBC load at pretreatment for ChemoRT can be applied to identify patients at risk for unfavorable survival due to high WBC load coupled with elevated PDL-1 staining. However, deeper exploration of these relationships and the tapering of steroids are important considerations in future studies and current approaches to managing GB patients.

## Figures and Tables

**Table 1:** Summarization of collected feature across study group and per site. Features listed as N/A were not collected at the site. Data is reported as either total (percent from collected patients) or mean [range].

Clinical Feature		Overall	OSU	UMMC	Barretos	FLENI
*N (%) or mean [range]*		N = 581	N = 377	N = 57	N = 94	N = 53
Age at Primary Surgery (years)		60.64 [20–89]	61.97 [20–89]	61.49 [25–80]	54.13 [29–76]	61.81 [20–78]
Gender	Male	342 (58.86%)	219 (58.09%)	36 (63.16%)	54 (57.45%)	33 (62.26%)
	Female	239 (41.14%)	158 (41.91%)	21 (36.84%)	40 (42.55%)	20 (37.74%)
Race	Native American	1 (0.17%)	0 (0%)	1 (1.75%)	0 (0%)	0 (0%)
	Asian	4 (0.69%)	4 (1.06%)	0 (0%)	0 (0%)	0 (0%)
	Black or African	30 (5.16%)	15 (3.98%)	15 (26.32%)	0 (0%)	0 (0%)
	Caucasian	467 (80.38%)	351 (93.10%)	40 (70.18%)	76 (80.85%)	0 (0%)
	More than One Race	14 (2.41%)	1 (0.27%)	0 (0%)	13 (13.83%)	0 (0%)
	Unknown	12 (2.07%)	6 (1.59%)	1 (1.75%)	5 (5.32%)	0 (0%)
	N/A	53 (9.12%)	0 (0%)	0 (0%)	0 (0%)	53 (100%)
Ethnicity	Hispanic/Latino	152 (26.16%)	5 (1.33%)	N/A	94 (100%)	53 (100%)
	Non-Hispanic/Latino	372 (64.03%)	372 (98.67%)	N/A	0 (0%)	0 (0%)
	N/A	57 (9.81%)	0 (0%)	57 (100%)	0 (0%)	0 (0%)
Vital Status	Alive	47 (8.09%)	23 (6.10%)	15 (26.32%)	4 (4.26%)	5 (9.43%)
	Deceased	530 (91.22%)	354 (93.90%)	42 (73.68%)	90 (95.74%)	44 (83.02%)
Overall Survival (days)		539.6 [31–3685]	560.4 [52–2787]	477.5 [45–3685]	533.8 [94–1471]	365.9 [31–930]
Age at Death (years)		62.39 [21–90]	64.08 [21–90]	N/A	56.07 [31–76]	61.61 [46–80]
Weight (kg)		87.29 [45–214]	87.29 [45–214]	N/A	N/A	N/A
Height (cm)		172.5 [147–196]	172.5 [147–196]	N/A	N/A	N/A
BMI		29.15 [17.8–66.1]	29.15 [17.8–66.1]	N/A	N/A	N/A
CCI Score		3.70 [0–11]	3.70 [0–11]	N/A	N/A	N/A
Lesion Side	Left	244 (42.0%)	197 (52.25%)	N/A	47 (50%)	N/A
	Right	213 (36.66%)	170 (45.09%)	N/A	43 (45.74%)	N/A
	Both	14 (2.41%)	10 (2.65%)	N/A	4 (4.26%)	N/A
	N/A	110 (18.93%)	0 (0%)	57 (100%)	0 (0%)	53 (100%)
Lesion Location	Frontal	163 (28.06%)	142 (37.67%)	N/A	21 (22.34%)	N/A
	Temporal	161 (27.71%)	119 (31.56%)	N/A	42 (44.68%)	N/A
	Parietal	97 (16.70%)	77 (20.42%)	N/A	20 (21.28%)	N/A
	Occipital	23 (3.96%)	22 (5.84%)	N/A	1 (1.07%)	N/A
	Brain Stem	14 (2.41%)	14 (3.71%)	N/A	0 (0%)	N/A
	Cerebellum	2 (0.34%)	2 (0.53%)	N/A	0 (0%)	N/A
	Mixed	11 (1.89%)	1 (0.27%)	N/A	10 (10.64%)	N/A
	N/A	110 (18.93%)	0 (0%)	57 (100%)	0 (0%)	53 (100%)
Lesion Size (cm)		4.34 [0.1–9.5]	4.18 [0.1–9.2]	N/A	N/A	N/A
Midline Shift	Yes	141 (24.27%)	141 (37.40%)	N/A	N/A	N/A
	No	221 (38.04%)	221 (58.62%)	N/A	N/A	N/A
	N/A	219 (37.69%)	15 (3.98%)	57 (100%)	94 (100%)	53 (100%)
ATRX Status	Intact	427 (73.49%)	341 (90.45%)	N/A	86 (91.49%)	N/A
	Loss	9 (1.55%)	3 (0.80%)	N/A	6 (6.38%)	N/A
	N/A	145 (24.96%)	33 (8.75%)	57 (100%)	2 (2.13%)	53 (100%)
p53 Mutation (> 10% of Cells)	Negative	72 (12.39%)	72 (19.10%)	N/A	N/A	N/A
	Positive	256 (44.06%)	256 (67.90%)	N/A	N/A	N/A
	N/A	253 (43.55%)	49 (13.00%)	57 (100%)	94 (100%)	53 (100%)
Ki67 (%)		31.84 [4–95]	31.84 [4–95]	N/A	N/A	N/A
*EGFR* Amplification	Yes	173 (29.78%)	173 (45.89%)	N/A	N/A	N/A
	No	136 (23.41%)	136 (36.07%)	N/A	N/A	N/A
	N/A	272 (46.82%)	68 (18.04%)	57 (100%)	94 (100%)	53 (100%)
*MGMT* Status	Hypermethylated	189 (32.53%)	155 (41.11%)	N/A	34 (36.17%)	N/A
	Unmethylated	249 (42.86%)	221 (58.62%)	N/A	28 (29.79%)	N/A
	N/A	143 (24.61%)	1 (0.27%)	57 (100%)	32 (34.04%)	53 (100%)
WBC Post-Surgical (×10^A^9/L)		9.78 [2.20–63.77]	9.96 [2.49–63.77]	9.76 [2.30–24.80]	8.72 [2.20–18.60]	10.57 [5.10–20.50]
Neutrophils Post-Surgical (×10^A^9/L)		7.21 [0.21–22.82]	7.37 [1.00–21.50]	7.26 [0.21–22.82]	6.02 [0.97–16.00]	8.53 [2.35–18.25]
Lymphocytes Post-Surgical (×10^A^9/L)		1.65 [0.07–54.2]	1.65 [0.08–54.2]	1.59 [0.07–4.68]	1.80 [0.12–5.60]	1.38 [0.38–3.54]
Neutrophil: Lymphocyte Ratio Post-Surgical		7.51 [0.06–137.90]	8.00 [0.06–115.13]	6.84 [0.85–30.67]	5.53 [1.01–137.90]	9.19 [1.12–32]
Platelets Post-Surgical (×10^A^9/L)		236.5 [43–593]	231.6 [64–593]	246.2 [43–522]	247.9 [84–467]	N/A
Steroids Post-Surgical (mg/day)		2.84 [0–24]	2.67 [0–24]	0.31 [0–2]	N/A	6.87 [0–16]
Radiation Dose (Gy)		55.59 [5.34–75]	55.82 [5.34–75]	N/A	54.66 [25–66]	N/A
Radiation Fractions		26.8 [1–50]	26.74 [1–50]	N/A	27.07 [5–33]	N/A
ChemoRT Treatment Time (Days)		42.2 [0–336]	38.14 [0–90]	N/A	62.09 [0–336]	N/A
Adjuvant Temozolomide Dose (mg)		238.1 [0–500]	238.1 [0–500]	N/A	N/A	N/A
Adjuvant Temozolomide Cycles		3.00 [0–19]	3.072 [0–19]	N/A	2.70 [1–12]	N/A
Time to Enhancement (days)		278.6 [41–2037]	278.6 [41–2037]	N/A	N/A	N/A
Enhancement Status	Recurrent	241 (41.48%)	241 (63.94%)	N/A	N/A	N/A
	Reactive	104 (17.90%)	104 (27.59%)	N/A	N/A	N/A
	Stable	32 (5.51%)	32 (8.49%)	N/A	N/A	N/A
	N/A	204 (35.11%)	0 (0%)	57 (100%)	94 (100%)	53 (100%)

**Table 2:** Univariate cox model results. Influence of clinical features to overall survival was evaluated using univariate cox regression. Features indirectly related to OS were omitted. Significant features are marked by asterisk.

Feature	Beta	HR (95% CI)	Wald Test	P-value
*Age at Surgery*	0.01944	1.02 (1.011–1.028)	21	4.20E-06*
*Gender*	−0.01552	0.9846 (0.8232–1.178)	0.03	0.87
*Race*	−0.002198	0.9978 (0.9908–1.005)	0.37	0.54
*Ethnicity*	0.1583	1.172 (0.9444–1.453)	2.1	0.15
*Weight*	0.002215	1.002 (0.9979–1.007)	0.99	0.32
*Height*	0.004985	1.005 (0.9954–1.015)	1	0.31
*BMI*	0.004967	1.005 (0.9892–1.021)	0.38	0.54
*CCI Score*	0.05545	1.057 (1.013–1.103)	6.5	0.011*
*Lesion Side*	0.03169	1.032 (0.868–1.228)	0.13	0.72
*Lesion Lobe*	−0.01187	0.9882 (0.913–1.07)	0.09	0.77
*ATRX*	0.1074	1.113 (0.5269–2.353)	0.08	0.78
*p53*	−0.1035	0.9017 (0.6851–1.187)	0.55	0.46
*Ki67*	0.00311	1.003 (0.9966–1.01)	0.88	0.35
*EGFR*	−0.2294	0.795 (0.6275–1.007)	3.6	0.057
*MGMT*	−0.4272	0.6523 (0.533–0.7983)	17	3.40E-05*
*Lesion Size*	0.01855	1.019 (0.9616–1.079)	0.4	0.53
*Midline Shift*	−0.1176	0.8891 (0.7115–1.111)	1.1	0.3
*WBC*	0.02951	1.03 (1.013–1.047)	13	0.00036*
*Platelets*	0.000202	1 (0.9991–1.001)	0.13	0.72
*Neutrophils*	0.04722	1.048 (1.023–1.074)	14	0.00015*
*Lymphocytes*	0.01214	1.012 (0.9767–1.049)	0.44	0.51
*NLR*	0.01261	1.013 (1.005–1.021)	9.3	0.0023*
*Steroids*	0.02151	1.022 (0.9942–1.05)	2.4	0.12
*Radiation Dose*	−0.05025	0.951 (0.9413–0.9608)	92	9.80E-22*
*Radiation Fractions*	−0.05064	0.9506 (0.9373–0.9641)	50	1.70E-12*
*Radiation Time*	−0.003487	0.9965 (0.9931–1)	3.9	0.049*
*TMZ Cycles*	−0.1612	0.8511 (0.8216–0.8816)	80	3.30E-19*
*TMZ Dose*	−0.00238	0.9976 (0.9969–0.9984)	41	1.80E-10*
*Center*	−0.05162	0.9497 (0.8576–1.052)	0.98	0.32

**Table 3:** Multivariate cox model results. Influence of clinical features to OS was evaluated using multivariate cox regression from features in Table 3. Significant features are marked by asterisk.

Feature	Beta	HR (95% CI)	P-value
**Age at Surgery**	0.02665	1.027 (1.011–1.044)	0.0011*
**CCI Score**	−0.01702	0.9831 (0.9198–1.051)	0.62
**MGMT**	−0.4308	0.65 (0.4904–0.8615)	0.0027*
**WBC**	0.01937	1.02 (0.9909–1.049)	0.18
**Neutrophils**	0.062	1.064 (1.013–1.117)	0.013*
**NLR**	−0.01111	0.989 (0.975–1.003)	0.13
**Radiation Dose**	−0.05578	0.9457 (0.9233–0.9687)	5.30E-06*
**Radiation Fractions**	0.0134	1.013 (0.9697–1.059)	0.55
**Radiation Time**	0.005447	1.005 (0.9876–1.024)	0.55
**TMZ Cycles**	−0.205	0.8146 (0.7653–0.8671)	1.20E-10*
**TMZ Dose**	0.0003521	1 (0.9993–1.001)	0.53

## Data Availability

The datasets used and/or analyzed during the current study are available from the corresponding authors on reasonable request. Dissemination of the applied REDCap form can be found in supplement or supplied from corresponding authors.
